# Radiation-induced thoracic necrosis with a pulmonary cutaneous fistula repaired using a free omental flap: a case report

**DOI:** 10.1186/s12893-019-0479-7

**Published:** 2019-02-02

**Authors:** Ryuichi Azuma, Masahito Kajita, Satoshi Kubo, Tomoharu Kiyosawa

**Affiliations:** Department of Plastic Surgery, National Defense Medical College, 3-2 Namiki Tokorozawa, Saitama, 359-0042 Japan

**Keywords:** Radiation therapy, Free omental flap, Pulmonary cutaneous fistula

## Abstract

**Background:**

Chest wall necrosis can manifest as a late effect of radiation therapy for breast cancer. Only two cases of fistulas communicating with the respiratory tract as a result of radiation-induced necrosis of the lungs or bronchi have been reported. To the best of our knowledge, we report the first case of a pulmonary cutaneous fistula arising as a late effect of radiation therapy for breast cancer, which was successfully repaired using a free omental graft.

**Case presentation:**

A 64-year-old woman underwent Halsted surgery and postoperative radiation therapy for breast cancer 25 years earlier. One year before visiting our hospital, she developed a fistula and bleeding in her left clavicular region, which was expanding. On initial examination, a 6-cm-wide skin defect was observed in the left clavicular region and the clavicle appeared sequestrated. Computed tomography revealed part of the first to third left ribs, part of the left clavicle, the subclavian artery, and the brachial plexus to be missing. Several rounds of debridement revealed approximately 10 bronchial stumps on the surface of the collapsed lung, from which exhaled air and sputum were effusing. Surgery was performed to implant a free omental flap with vascular anastomosis and a skin graft in the neck region, and the pulmonary cutaneous fistula was closed. Two years after surgery, emphysema remained inside the omentum, which spontaneously resolved by the 3rd postoperative year.

**Conclusions:**

Various treatment options are conceivable for the repair of pulmonary cutaneous and bronchocutaneous fistulas induced by radiation damage (e.g., free tissue grafts and endoscopic bronchial occlusion); however, these are rarely reported, and the most reliable method thus remains unclear. Positive outcomes in our case indicate that implanting a free omental graft may be effective. Furthermore, spontaneous healing can be expected for the residual emphysema inside the omentum.

## Background

Necrosis of the chest wall can manifest as a late effect of radiation therapy for breast cancer [1, 2]. In majority of the cases, necrosis occurs in the skin and cartilage, and only two cases of fistulas communicating with the respiratory tract as a result of radiation-induced necrosis of the lungs or bronchi have been reported to date [3, 4]. However, repair of a pulmonary cutaneous or bronchocutaneous fistula using a free tissue graft has not been reported. To the best of our knowledge, we report the first case of a pulmonary cutaneous fistula arising as a late effect of radiation therapy for breast cancer treatment administered 25 years previously, which was successfully repaired using a free omental graft.

## Case presentation

A 64-year-old woman was referred to our hospital for the treatment of chest wall necrosis. She had undergone Halsted surgery and postoperative radiation therapy with cobalt-60 (30 Gray) and megavoltage X-rays (30 Gray) 25 years earlier. Following the treatment, her left upper limb became completely paralyzed.

Six years earlier, she had sustained a left clavicle fracture due to osteonecrosis, and she had subsequently developed a chronic cutaneous fistula measuring 1 cm in diameter. The fracture and cutaneous fistula failed to heal, and she experienced bleeding from the fistula since 1 year. Two months before visiting our hospital, she was hospitalized in the Department of Breast Surgery at another hospital for massive bleeding and local infection. She was treated with antibiotics and cleansing of the fistula and the infection subsided 2 months thereafter. She visited our hospital after discharge.

**Fig. 1:**
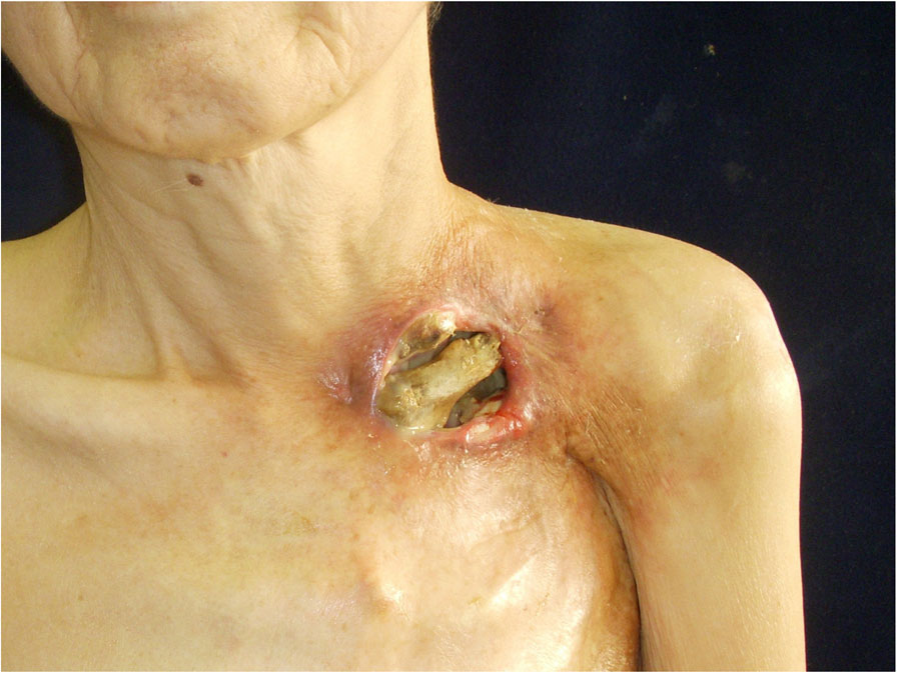
Findings of the initial examination. Necrosed left clavicle was exposed, and air leaked from deep within the chest. The upper left limb showed marked ischemia and was completely paralyzed

**Fig. 2:**
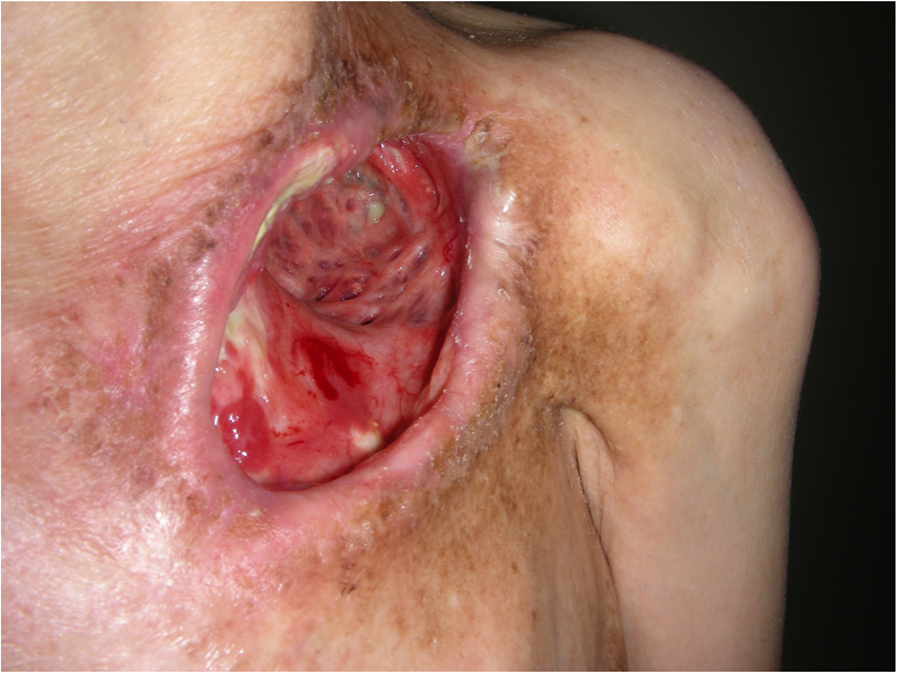
Preoperative findings. Necrosed parts of the left clavicle and the first and second ribs were removed. Approximately 10 exposed bronchial and bronchiolar stumps were observed on the surface of the lung, which were visible at the base of the defect

At initial examination, her height was 153 cm and weight was 34 kg. Necrosed clavicle was exposed through the 6-cm-wide skin defect in the left clavicular region, and air entered and exited the opening from deep within the chest while breathing (Fig. 1). Computed tomography (CT) revealed the part of the first to third left ribs, part of the left clavicle, the subclavian artery, and the brachial plexus to be missing. Her upper left limb was nourished by retrograde blood flow from the thoracodorsal artery; however, blood flow was weak to the skin of the arm, the surface of which was cold, resulting in complete paralysis of the upper extremity. Several rounds of debridement eliminated the necrotic tissue, and the local infection was completely resolved. Debridement revealed a tissue defect measuring 4 cm × 8 cm and 4 cm deep at the base and approximately 10 bronchial stumps on the surface of the collapsed lung covered in granulation tissue, from which exhaled air and sputum were effusing (Fig. 2). She presented with severe emaciation and malnutrition, but no other systemic anomalies were noted.

**Fig. 3:**
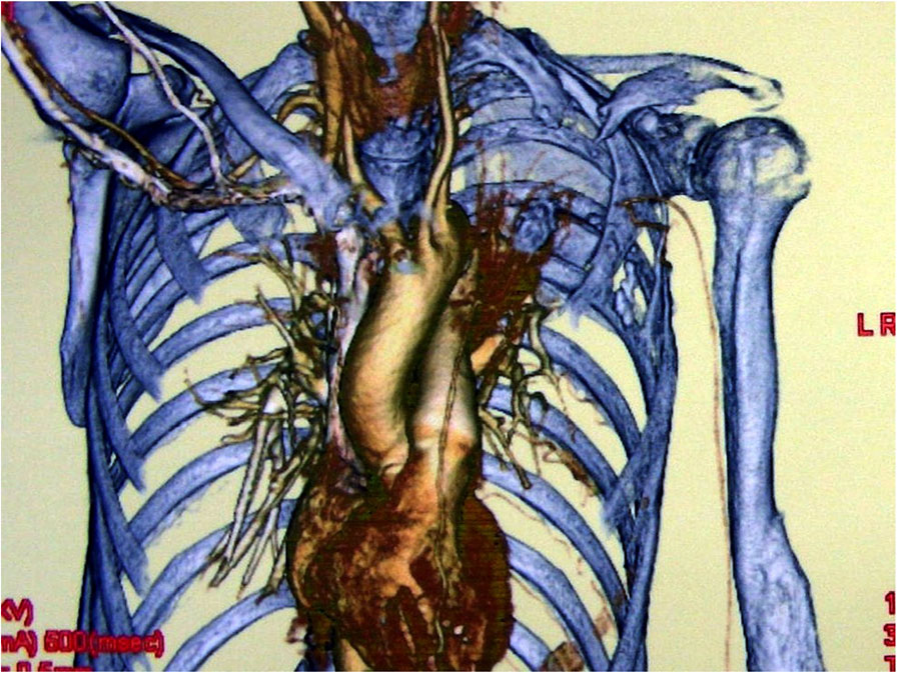
Preoperative computed tomography angiography. The subclavian artery was missing, and blood flow to the brachial artery was retrograde via the thoracodorsal artery to the subscapular artery

After treatment, she had almost no problem with daily life but frequently coughed while taking baths or when exposed to cold air. Therefore, she consented to undergo the surgical closure of the pulmonary fistula. A left latissimus myocutaneous flap could not be used as the reconstructive material due to the absence of vascular pedicle (Fig. 3). A free rectus abdominis flap was considered, but an omental flap was ultimately selected for its perceived ability to create the best air seal. Owing to her severe emaciation, the volume of the omentum available for harvest was considered inadequate; therefore, she was administered a diet by a nutritionist for 9 months to improve her systemic nutritional state and thereby increase her resistance. Subsequently, her weight increased from 34 kg to 38 kg, and her serum albumin level improved from 3.0 g/dL to 3.7 g/dL. Reconstructive surgery was performed. Preoperative blood gas analysis revealed pH of 7.400, carbon dioxide partial pressure of 45.3 mmHg, and oxygen partial pressure of 84.3 mmHg. Lung function test performed while holding down the pulmonary fistula revealed forced vital capacity of 1.16 L and forced expiratory volume as 1% of 100%.

### Intraoperative findings

Surgery was performed under general anesthesia with selective one lung ventilation.

**Fig. 4:**
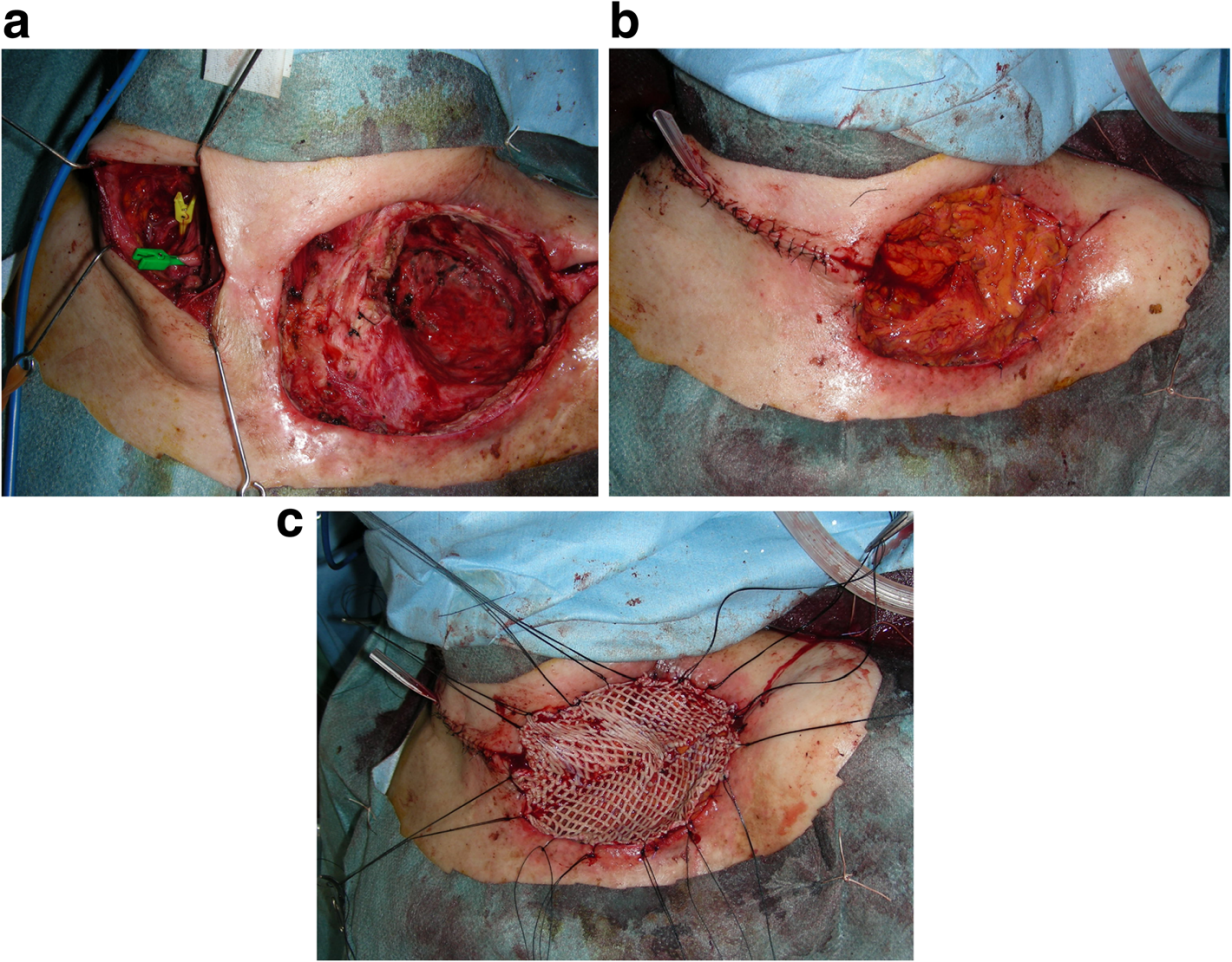
Intraoperative findings. **a**: The skin, necrosed bone, and other structures surrounding the defect were removed. The right transverse cervical artery and right anterior jugular vein were exposed due to vascular anastomosis. **b**: Following vascular anastomosis, the tissue defect was filled with the omentum. The intermediate skin bridge over the pedicle was incised and resutured. **c**: A 20/1000-in.-thick split-thickness skin graft was placed over the omentum from the abdomen

The damaged skin, clavicle, and ribs surrounding the defect were debrided. The base of the defect was covered with hemorrhagic granulation tissue and was only rubbed with gauze for debridement to avoid over expansion of the bronchial stumps. A free omental flap (approximately 155 cc) with the right gastroepiploic blood vessel as its pedicle was harvested under laparoscopy. The right transverse cervical artery and right anterior jugular vein were anastomosed with the right gastroepiploic blood vessel; the tissue defect was filled with the omentum. A 20/1000-in.-thick split-thickness skin graft was placed over the omentum, and the graft was immobilized with tie-over dressing (Fig. 4).

### Postoperative course

**Fig. 5:**
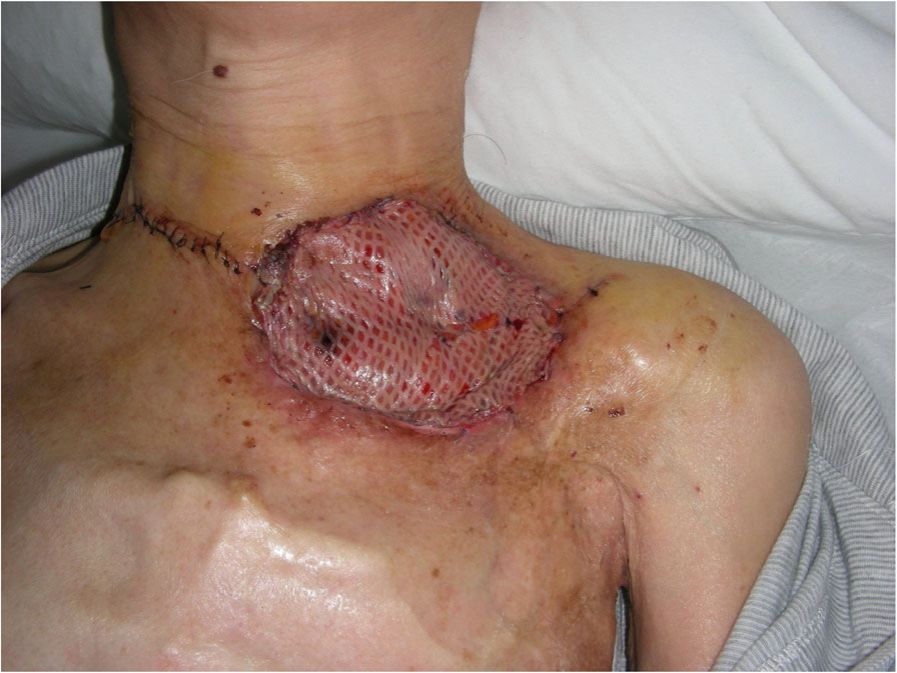
Findings at postoperative day 5. The skin graft survived, and air leakage stopped

**Fig. 6:**
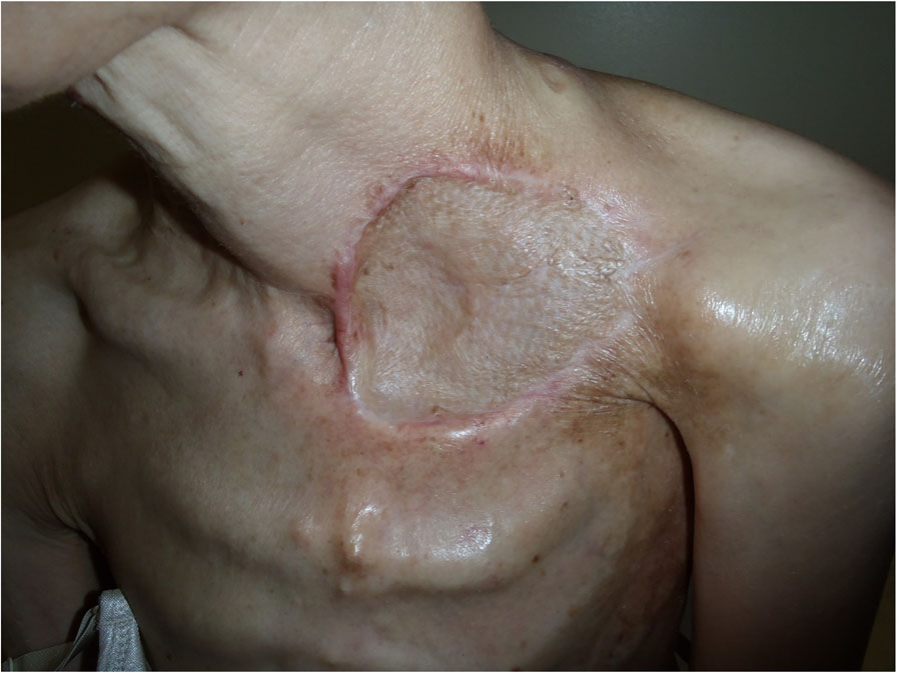
Findings at 1 year after surgery. The pulmonary cutaneous fistula was closed, but emphysema was noted inside the omentum. The skin graft moved while breathing

**Fig. 7:**
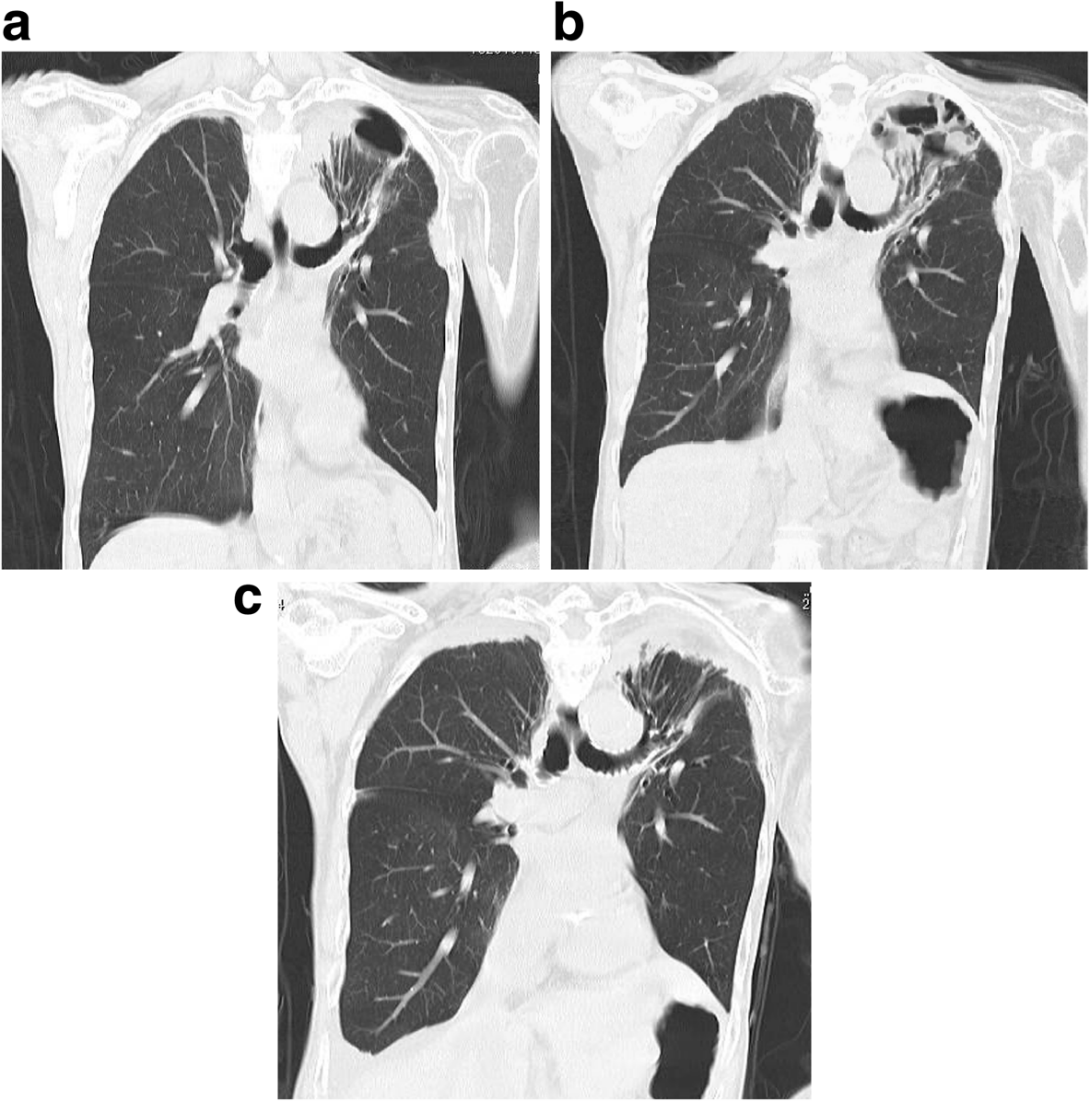
Computed tomography scans before the surgery and at 2 and 7 years after the surgery. **a**: The upper left lobe collapsed, and the peripheral B1/2 and B3 bronchi or bronchioles were open at the base of the fistula. **b**: Emphysema was visible inside the omental filling, but there was no communication between emphysema and the skin surface. **c**: Emphysema resolved. The grafted omentum and surrounding scar tissue were visible

The tie-over dressing was removed on the 5th postoperative day, and the engraftment of the omentum and skin graft was observed to ensure that there was no air leakage (Fig. 5). However, a bulge that moved with breathing was noted on the skin graft over the omentum 2 weeks after surgery. CT confirmed this bulge to be the buildup of air between the transplanted omental tissues (omental emphysema). Pressure was applied to the bulge with scrunched up gauze and adhesive tape for 2 months; however, omental emphysema persisted even after 2 months; therefore, the dressing was removed, and she instructed to apply pressure with the hand when coughing. She is currently undergoing the follow-up and has not developed any subsequent emphysematous or respiratory infection (e.g., pneumonia). Omental emphysema persisted almost entirely without change for at least 2 years after surgery, but it spontaneously resolved by the 3rd postoperative year (Fig. 6). Changes over time, as observed in CT, at 7 years after the surgery are presented in Fig. 7.

## Discussion and conclusions

Overall, 2–12% of the patients who undergo radiation therapy for breast or lung cancer experience damage to the skin or thorax [5]. When this damage is mild, radiation dermatitis or skin ulcers occur; however, more severe damage can result in pleurocutaneous fistulas due to necrosis of the thorax, including the clavicle and ribs. In our case, the patient presented with additional pulmonary necrosis, which exposed the bronchial and bronchiolar stumps, leading to the development of a pulmonary cutaneous fistula.

The damaged area comprised the clavicular region, for which reconstruction using a pedicled latissimus dorsi myocutaneous flap is typically the first choice [6]. However, subclavian blood vessels were disrupted in our patient, resulting in a barely functional left upper limb due to retrograde blood flow from the branch of the thoracodorsal artery. Therefore, the use of a latissimus dorsi flap was considered impossible (Fig. 3).

Consequently, we decided to use a free tissue graft and selected a free omental graft. Reconstruction of radiation-induced tissue necrosis using muscle flaps [7–10] and the omentum has been reported [7–9, 11–14]. Owing to the biological properties of the omentum, Van Geel et al. [13] have discussed the utility of omental grafts for chest wall defects caused by radiation damage. Moreover, other reports have supported the utility of omental grafts in the closure of bronchopleural fistulas following surgery for lung cancer [15, 16]. In terms of the sealing of air leaks from bronchial stumps, the use of various autologous tissues, such as pericardial fat pads, the pleura, and the diaphragm, besides suturing [17], has been reported. For the treatment of bronchopleural fistulas caused by lung cancer surgery, fungal infections, or tuberculosis, O’Neill et al. [4] have reported the efficacy of endobronchial balloon occlusion and selective endobronchial intubation, while Watanabe et al. [18] have reported the use of endoscopical plugging and closing of the bronchial tube with a silicone stopper (Endoscopic Watanabe Spigot). Although we have no experience of plugging bronchial stumps using a specialized device, these methods may guarantee the closure of bronchial stumps.

In the present case, however, when reconstructive surgery was planned, an emphysematous infection, which can lead to the development of pneumonia, sepsis, and potential death, could have occurred in case the closure of the bronchial stump had failed. Despite the development of emphysema in our case, she did not develop any infection, and her emphysema resolved 3 years after surgery.

Various conceivable treatment options exist for the reconstruction of pulmonary cutaneous and bronchocutaneous fistulas caused by radiation damage, including free tissue grafts and endoscopic bronchial occlusion. However, these approaches are rarely reported, and the most reliable method thus remains unclear. In the present case, positive outcomes obtained using the combination of omental and skin grafts highlight the effectiveness of this approach. Furthermore, spontaneous healing can be expected for emphysema within the omentum.

## Abbreviations

CT: computed tomography
